# Bone Mineral Density Is Positively Related to Carotid Intima‐Media Thickness: Findings From a Population‐Based Study in Adolescents and Premenopausal Women

**DOI:** 10.1002/jbmr.2903

**Published:** 2016-09-01

**Authors:** Monika Frysz, Kevin Deere, Debbie A Lawlor, Li Benfield, Jon H Tobias, Celia L Gregson

**Affiliations:** ^1^ School of Social and Community MedicineUniversity of BristolMRC Integrative Epidemiology Unit at the University of BristolBristolUK; ^2^ Musculoskeletal Research UnitSchool of Clinical SciencesUniversity of BristolBristolUK; ^3^ School of Social and Community MedicineUniversity of BristolBristolUK

**Keywords:** ATHEROSCLEROSIS, CARDIOVASCULAR DISEASE, OSTEOPOROSIS, BONE MINERAL DENSITY, ALSPAC

## Abstract

Osteoporosis and cardiovascular disease (CVD) are both common causes of morbidity and mortality. Previous studies, mainly of people older than 60 years, suggest a relationship between these conditions. Our aim was to determine the association between bone characteristics and CVD markers in younger and middle‐aged individuals. Women (*n* = 3366) and their adolescent offspring (*n* = 4368) from the UK population‐based cohort study, Avon Longitudinal Study of Parents and Children (ALSPAC), were investigated. We measured total body (TB) and hip bone mineral density (BMD), TB bone area (BA) and bone mineral content (BMC) by dual‐energy X‐ray absorptiometry (DXA), and carotid intima‐media thickness (cIMT) by high‐resolution ultrasound. Arterial distensibility was calculated as the difference between systolic and diastolic arterial diameters. Linear regression determined associations between bone exposures and cIMT (in adolescents) and both cIMT and arterial distensibility (in women), generating partial correlation coefficients. Mean (SD) age of women was 48 (4.2) years, body mass index (BMI) was 26.2 (5.0) kg/m^2^, and 71% were premenopausal. In confounder‐adjusted analyses (age, height, lean mass, fat mass, menopause, smoking, estrogen replacement, calcium/vitamin D supplementation, and education) TB and hip BMD were both positively associated with cIMT (0.071 [0.030, 0.112], *p* = 0.001; 0.063 [0.025, 0.101], *p* = 0.001, respectively). Femoral neck BMD and TB BMD, BMC, and BA were positively associated with arterial distensibility. Mean (SD) age of adolescents was 17 (0.4) years, BMI was 23 (4.1) kg/m^2^, and 44.5% were male. Total hip and TB measurements were positively associated with cIMT, with similar magnitudes of association to those found in their mothers. In contrast to most published findings, we identified weak positive associations between BMD and cIMT in predominantly premenopausal women and their adolescent offspring. We found greater femoral neck BMD and TB DXA measurements to be associated with reduced arterial stiffness. Rather than a relationship with preclinical atherosclerosis, in these relatively young populations, we speculate our associations between BMD, cIMT, and arterial distensibility may reflect a shared relationship between bone and vascular growth and development. © 2016 The Authors. *Journal of Bone and Mineral Research* published by Wiley Periodicals, Inc. on behalf of American Society for Bone and Mineral Research.

## Introduction

Osteoporosis and cardiovascular disease (CVD) are both common age‐related conditions associated with increased morbidity and mortality. Within aging populations, the prevalence of both is rising, with major socioeconomic and health consequences.^1^ Several cross‐sectional and prospective studies have examined the association between CVD and bone density or related bone outcomes, and a recent systematic review of 43 cross‐sectional and 27 prospective studies concluded that people with CVD are at risk of bone loss and subsequent fractures.^2^ Atherosclerosis, which underlies CVD, is a chronic inflammatory process that begins in young adulthood,^3^ and studies of markers of preclinical atherosclerosis, as measured by carotid intima‐media thickness (cIMT), further suggest greater atherosclerotic risk is associated with lower bone mineral density (BMD).^4^, ^5^ Human arteries consist of three layers: the innermost layer being the intima, then the media, and, on the outside of the vessel, the adventitia; cIMT is the distance between the lumen‐intima and the media‐adventitia interfaces. The intima consists of endothelium, the media is composed of smooth muscle cells, whereas the adventitia is mainly composed of elastic and collagen fibers.^6^ Similarly, the unmineralized organic component of bone, the osteoid, is also composed of collagen along with other components such as matrix vesicles, osteopontin, and other non‐collagenous bone matrix proteins.^7^ Whereas symptomatic CVD might result in reduced physical activity, and sedentary behavior could then lead to reductions in BMD, this in isolation is unlikely to explain associations between cIMT and BMD or osteoporotic outcomes. Most participants in the studies undertaken to date have been aged 60 years or older. Although all adjusted for age, it is difficult in studies where all participants are older to fully control for all age‐related characteristics that might produce a non‐causal (confounded) association between CVD and BMD. Furthermore, given lack of data from younger populations, it is unknown whether associations reported in older populations generalize to younger groups. We were, therefore, interested in whether similar associations are found in younger populations. We hypothesized that preclinical atherosclerosis (measured by cIMT) would be associated with lower BMD even in younger populations than had hitherto been studied.

The aim of this study was to determine the association between bone characteristics (i.e., hip and total body BMD) and preclinical atherosclerosis risk in young and middle‐aged people. Preclinical atherosclerosis was assessed by cIMT (a widely accepted marker of atherosclerosis and predictor of future vascular events^8^), and, in the middle‐aged women only, arterial distensibility (stiffness).

## Materials and Methods

### Study participants

Data from the mothers and their adolescent offspring in the Avon Longitudinal Study of Parents and Children (ALSPAC) were used. ALSPAC is a longitudinal birth cohort that recruited pregnant women resident in a geographical area in the South West of England, UK, with an expected delivery date between April 1, 1991, and December 31, 1992.^9^, ^10^ The study website contains details of all available data through a fully searchable data dictionary (http://www.bris.ac.uk/alspac/researchers/data-access/data-dictionary/). In total, 14,541 pregnancies were initially enrolled (for details, see www.alspac.bris.ac.uk), 674 were excluded (604 were non‐live births, 69 unknown outcomes, and 1 live birth from a twin pregnancy); the remaining pregnancies included 13,761 individual women. The present study uses data from follow‐up research clinics of the mothers and adolescents that were both undertaken between 2008 and 2011. All eligible mothers and their offspring (i.e., still engaged with the study; alive with known contact details and who had not withdrawn their consent) were invited to these assessments. Of 11,264 (82%) women invited, 4834 (43%) attended. Of 10,101 (65%) adolescents invited, 5217 (52%) attended (Supplemental Fig. S1 illustrates participant recruitment). Full study details have previously been reported.^9^, ^11^ Our study is cross‐sectional, nested within the ongoing ALSPAC cohort.

Written informed consent was collected for all in line with the Declaration of Helsinki.^12^ Ethical approval for the study was obtained from the ALSPAC Ethics and Law Committee and the Local Research Ethics Committees (North Somerset and South Bristol Research Ethics Committee: 08/H0106/96).

### Exposure measurements

Dual‐energy X‐ray absorptiometry (DXA) scans, performed by GE Lunar Prodigy (Madison, WI, USA), were acquired and analyzed according to the manufacturer's standard scanning and positioning protocols. Total body less head BMD (g/cm^2^), bone mineral content (BMC) (g), and bone area (BA) (cm^2^) were measured, together with total body (TB) fat mass and lean mass (kg), total hip, femoral neck, and trochanter BMD (g/cm^2^) and total hip BMC (g), plus derived hip cross‐sectional moment of inertia (CSMI, cm^4^). Further details, including reproducibility, have been described previously;^13^ e.g., coefficient of variation for TB BMD was 0.8%.

### Outcome measurements

cIMT (mm) scans of the left and right common carotid arteries were obtained via high‐resolution B ultrasound using a standardized protocol.^14^ This tool is a valid and strong predictor of CVD risk (e.g., Atherosclerosis Risk in Communities;^15^ the Rotterdam study^14^). Both arteries were imaged longitudinally 1 cm proximal to the carotid bifurcation using a ZONARE z.one Ultra convertible ultrasound system with L10‐5 linear transducer. Images focused on the posterior (far) arterial wall using the zoom magnifying function. Ten‐second cine loops were recorded in DICOM format and analyzed offline using Carotid Analyzer for Research (Vascular Research Tools 5, Medical Imaging Applications, LLC, Iowa City, IA, USA, 2008). Three end‐diastolic frames from three consecutive cardiac cycles were recorded for left and right carotid arteries, and the mean across left and right readings was used in analyses. Arterial distensibility was calculated as the difference between systolic and diastolic arterial diameters; the mean of left and right readings was used. Images were analyzed by a single trained reader.

### Confounding variables

We considered the following to be potential confounders: age, fat mass, lean mass, height, socio‐economic position (SEP), tobacco smoking, and alcohol consumption. In addition, further confounding variables were considered for the women: menopausal status, hormone‐replacement use, educational attainment, daily calcium intake, calcium and/or vitamin D supplement use, and levels of self‐reported physical activity (PA). Participants completed a series of questionnaires to collect these data. Women were considered postmenopausal if they had not had a period for 12 months or if their periods had stopped after hysterectomy, ablation or resection, chemotherapy, or radiation therapy. Women were specifically asked about hormone‐replacement use and details of all (over‐the‐counter and prescribed) medications, including calcium and/or vitamin D supplementation. Household occupations and highest educational qualification were obtained from a questionnaire completed during the index pregnancy. Highest occupation was used to define social class using the 1991 British Office of Population Censuses and Surveys classification.^16^ Educational attainment was dichotomized as having a university degree or not. Mothers’ self‐reported smoking, alcohol consumption, and PA were obtained from a mailed questionnaire completed in 2010. The PA questionnaire is similar to that used in the British Women's Heart and Health study^17^ and British Regional Heart Study^18^ and has been validated against resting heart rate.^19^ Data from a food‐frequency questionnaire (completed in 2004) was used to estimate daily calcium intake (mg).^20^ Height was measured to the nearest 0.1 cm using a Harpender stadiometer without shoes. Smoking and alcohol consumption data for the adolescents were collected via a computer‐based questionnaire in clinic.

### Statistical methods

The normality of data was explored using descriptive statistics and histograms. Fat and lean mass in mothers and fat mass in adolescents were positively skewed so they were log transformed for analysis. Descriptive statistics are expressed as means with standard deviations (SD) for continuous variables and counts with percentages (%) for categorical variables. All associations were analyzed using multivariable linear regression, and results expressed as partial correlation coefficients (β) with 95% confidence intervals (i.e., SD change in dependent variable per SD increase in exposure). We examined the associations of all bone exposures with cIMT and arterial distensibility (adult women only) in a series of models. In women: model 1 was unadjusted; model 2 adjusted for age; model 3 additionally adjusted for height, lean mass, fat mass, menopause, smoking, hormone replacement, calcium and/or vitamin D supplement use, and education. In adolescents: model 1 was unadjusted; model 2 age and sex adjusted; model 3 was additionally adjusted for height and lean and fat mass. We examined an unadjusted and then age only (and age and sex in adolescents) adjusted models as we felt age was likely to be the major confounding factor and wanted to determine the extent to which it influenced associations. In adolescent analyses, we explored sex differences by comparing results (regression coefficients and their 95% confidence intervals) between males and females and by testing for evidence of a statistical interaction between sex and the exposures in relation to the associations with cIMT.

The main analyses, as described above, were conducted on 3366 women with complete data on outcome variables, exposures, and most confounders. Of 3366 women, 1936 (57%) had data for alcohol consumption, PA level, calcium intake, and SEP. We conducted a sensitivity analysis in this subgroup examining whether further adjustment (in addition to model 3 described above) for these four additional potential confounders influenced our findings. Because the relationship between osteoporosis and atherosclerosis may differ before and after menopause, we conducted a further sensitivity analysis excluding postmenopausal women, repeating the main analyses in the subgroup of 2382 (71%) premenopausal women.

The main analyses were conducted in 4368 adolescents. We conducted a sensitivity analysis in the subgroup of 2836 (65%) with additional data for alcohol consumption, smoking, and SEP to examine whether further adjustment (in addition to model 3 described above) for these three additional potential confounders influenced our findings. All statistical analyses were performed using Stata version 13.1 (StataCorp, College Station, TX, USA).

## Results

### Adult women

Table 1 shows the characteristics of the 3366 women included in this study. Their mean (SD) age was 48 (4.2) years, with BMI 26.3 (5.1) kg/m^2^; 71% were premenopausal with 5.2% using hormone‐replacement therapy. The majority (86.8%) had a total hip *T*‐score >–1.0; only 0.5% had a *T*‐score <–2.5. In comparison with the 10,395 women excluded because of non‐clinic attendance or missing data, participating women were older, more likely to have a university education and own property, have had lower prepregnancy BMI, and were less likely to smoke (Supplemental Table S1).

**Table 1:** Characteristics of Participating ALSPAC Cohort Study Women

	*n*	Mean (SD) for continuous variables and prevalence *n* (%) for categorical variables
Exposure measurements		
Total hip BMD (g/cm^2^)	3366	1.03 (0.14)
TB BMD minus head (g/cm^2^)	3366	1.09 (0.08)
TB BMC minus head (g)	3366	2164.7 (365.8)
TB area minus head (cm^2^)	3366	1972.9 (231.1)
Outcome measurements		
cIMT (mm)	3366	0.56 (0.06)
Average arterial distensibility (mm)	3366	0.50 (0.12)
Confounder variables		
Age (years)	3366	48.1 (4.2)
Height (cm)	3366	164.2 (6.1)
Log_e_ fat mass (kg)	3366	3.2 (0.4)
Log_e_ lean mass (kg)	3366	3.7 (0.1)
Daily calcium intake (mg)^a^	1936	476.4 (180.0)
Calcium and/or vitamin D supplements [*n* (%)]	3366	100 (3.0)
Menopause [*n* (%)]	3366	984 (29.2)
Hormone replacement [*n* (%)]	3366	175 (5.2)
Current/ex‐smoker [*n* (%)]	3366	1,303 (38.7)
Physical activity [*n* (%)]^a^	1936	
None		98 (5.1)
Minimal/limited		236 (12.2)
Some		917 (47.4)
Moderate		402 (20.8)
Very active		283 (14.6)
Social class [*n* (%)]^a^	1936	
I		190 (9.8)
II		766 (39.6)
III (non‐manual)		750 (38.7)
III (manual)		92 (4.8)
IV/V		138 (7.1)
Frequency of alcohol consumption [*n* (%)]^a^	1936	
Never		160 (8.3)
Monthly or less		293 (15.1)
2 to 4 times/month		354 (18.3)
2 to 3 times/week		984 (35.3)
4 or more times/week		445 (23.0)
Other variables		
BMI	3366	26.3 (5.1)
Total hip *T*‐score between –1 and –2.5 SD [*n* (%)]^b^	3357	429 (12.8)
Total hip *T*‐score below –2.5 SD [*n* (%)]^b^	3357	15 (0.45)

SD = standard deviation; cIMT = common carotid intima‐media thickness; BMD = bone mineral density; BMC = bone mineral content.

Results are shown as mean (SD) for continuous variables and *n* (%) for categorical variables.

^a^ Valid data available only in a subgroup, *n* = 1936.

^b^ Valid data available only in a subgroup, *n* = 3357.

#### Association between hip and TB DXA measurements and cIMT (Table 2)

**Figure 1:**
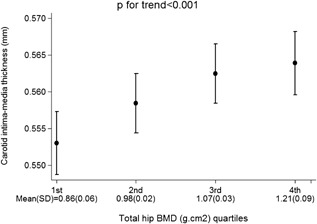
Adjusted mean carotid intima‐media thickness according to total hip BMD quartiles in 3366 study women. Mean (SD) for total hip BMD quartiles presented on *x* axis. Error bars represent 95% confidence intervals; *p* value shows test for trend.

Hip DXA measurements, including total hip, femoral neck, and trochanteric BMD, were all positively associated with cIMT (model 1), and these associations were strengthened after adjustment for age (model 2) but attenuated after further confounder adjustment (model 3) (Table 2). For example, in model 3, for every SD greater total hip BMD, cIMT was on average 0.03 mm thicker (95% confidence interval [CI] 0.011, 0.044; *p* = 0.001). For illustration purposes, total hip BMD was divided into quartiles. Figure 1 shows that successive quartile increases in total hip BMD were associated with greater cIMT. A positive association was found between hip strength (CSMI) and cIMT (model 1; 0.106 [0.072, 0.140]; *p* < 0.001), which was partially attenuated towards the null by full confounder adjustment (model 3; 0.052 [0.012, 0.092]; *p* = 0.011).

**Table 2:** The Association Between Hip and Total Body DXA Measurements and cIMT Among ALSPAC Study Women

	SD change in cIMT per 1 SD change in exposure (95% CI) (*n* = 3366)					
Exposure	Model 1	*p* Value	Model 2	*p* Value	Model 3	*p* Value
Total hip BMD	0.068 (0.034, 0.102)	<0.001	0.110 (0.077, 0.144)	<0.001	0.063 (0.025, 0.101)	0.001
Femoral neck BMD	0.057 (0.022, 0.091)	0.001	0.105 (0.071, 0.139)	<0.001	0.055 (0.018, 0.091)	0.004
Trochanter BMD	0.089 (0.055, 0.122)	<0.001	0.116 (0.083, 0.150)	<0.001	0.069 (0.032, 0.106)	<0.001
CSMI	0.106 (0.072, 0.140)	<0.001	0.118 (0.085, 0.151)	<0.001	0.052 (0.012, 0.092)	0.011
Total body BMD	0.084 (0.051, 0.119)	<0.001	0.132 (0.099, 0.165)	<0.001	0.071 (0.030, 0.112)	0.001
Total body BA	0.087 (0.053, 0.121)	<0.001	0.117 (0.084, 0.151)	<0.001	0.048 (–0.004, 0.100)	0.071
Total body BMC	0.097 (0.063, 0.131)	<0.001	0.139 (0.105, 0.172)	<0.001	0.076 (0.027, 0.125)	0.003
aBMC	0.124 (0.031, 0.217)	0.009	0.218 (0.128, 0.309)	<0.001	0.152 (0.051, 0.253)	0.003

cIMT = common carotid intima‐media thickness; CI = confidence interval; BMD = bone mineral density; CSMI = cross‐sectional moment of inertia (cm^4^); BA = bone area; BMC = bone mineral content; aBMC = area‐adjusted total body bone mineral content.

Table shows results of linear regression analysis between hip and total body DXA measurements and cIMT in 3366 individuals. Results are standard deviation change in cIMT per standard deviation increase in exposure (95% confidence intervals) and *p* value. Model 1 = unadjusted analysis; model 2 = adjustment for age; model 3 = model 2 plus additional adjustment for height, lean mass, fat mass, menopause, smoking, hormone replacement, calcium and/or vitamin D supplement use, and education.

**Figure 2:**
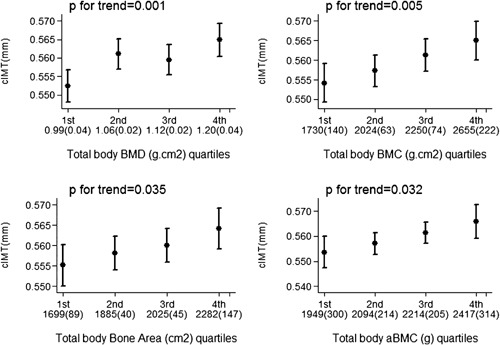
Adjusted mean carotid intima‐media thickness according to total body BMD, total body BMC, total bone area, and area‐adjusted total body BMC quartiles, in 3366 study women. Mean (SD) for each quartile presented on *x* axis. Error bars represent 95% confidence intervals; *p* values show test for trend.

Total body BMD, BMC, and BA were all positively associated with cIMT (model 1), and these associations were strengthened after adjustment for age (model 2) (Table 2). These associations were then partially attenuated by further adjustment in model 3. A positive association was found between area‐adjusted TB BMC and cIMT; this association was strengthened by adjustment for confounders (models 2 and 3). Successive total body BMD, BMC, BA, and aBMC (presented as quartiles for illustration purposes) were associated with greater cIMT (Fig. 2).

#### Association between hip and TB DXA measurements and arterial distensibility (Table 3)

Having identified positive relationships between bone parameters and cIMT, which were in the opposite direction to most epidemiological reports to date,^2^ we extended our analyses to assess further vascular properties, principally arterial wall stiffness. Each year of increasing age was associated with a 0.005 mm (95% CI 0.004, 0.006; *p* < 0.001) increase in arterial wall stiffness (i.e., reduced distensibility), as well as 0.003 mm (95% CI 0.003, 0.004; *p* < 0.001) in cIMT. cIMT and arterial distensibility were positively correlated (*r* = 0.17), such that without any adjustment, for each 1 mm increase in cIMT, distensibility increased by 0.32 mm (95% CI 0.25, 0.38; *p* < 0.001). After age adjustment, this relationship strengthened (β 0.42, 95% CI 0.36, 0.48; *p* < 0.001).

Hip DXA measurements, including femoral neck BMD and CSMI, were associated with reduced arterial wall stiffness (i.e., greater distensibility) (model 1) (Table 3). Femoral neck BMD and CSMI associations were partially attenuated after full confounder adjustment (model 3). After additional adjustment for cIMT (model 3 + cIMT) associations were only partially attenuated, suggesting associations with carotid‐intimal thickness do not fully account for those with arterial stiffness. Although total hip BMD was positively related to arterial distensibility (model 1; 0.037 [0.003, 0.071]; *p* = 0.031), this association was fully attenuated by age adjustment (model 2), and further adjustment for cIMT made little difference to these results.

**Table 3:** The Association Between Hip and Total Body DXA Measurements and Mean Arterial Distensibility Among ALSPAC Study Women

	SD change in average arterial distensibility per 1 SD change in exposure (95% CI) (*n* = 3366)							
Exposure	Model 1	*p* Value	Model 2	*p* Value	Model 3	*p* Value	Model 3 + cIMT	*p* Value
Total hip BMD	0.037 (0.003, 0.071)	0.031	0.005 (–0.029, 0.039)	0.769	0.034 (–0.003, 0.072)	0.072	0.021 (–0.016, 0.057)	0.270
Femoral neck BMD	0.077 (0.043, 0.110)	<0.001	0.041 (0.007, 0.075)	0.017	0.054 (0.018, 0.091)	0.004	0.042 (0.007, 0.078)	0.020
Trochanter BMD	0.029 (–0.004, 0.063)	0.090	0.007 (–0.026, 0.040)	0.670	0.032 (–0.006, 0.069)	0.097	0.016 (–0.020, 0.053)	0.376
CSMI	0.094 (0.060, 0.127)	<0.001	0.084 (0.052, 0.117)	<0.001	0.068 (0.028, 0.108)	0.001	0.057 (0.018, 0.096)	0.004
Total body BMD	0.074 (0.041, 0.107)	<0.001	0.040 (0.007, 0.073)	0.019	0.062 (0.021, 0.102)	0.003	0.046 (0.007, 0.086)	0.022
Total body BA	0.094 (0.060, 0.127)	<0.001	0.071 (0.038, 0.104)	<0.001	0.121 (0.070, 0.173)	<0.001	0.111 (0.061, 0.161)	<0.001
Total body BMC	0.096 (0.062, 0.129)	<0.001	0.066 (0.030, 0.099)	<0.001	0.113 (0.064, 0.162)	<0.001	0.097 (0.049, 0.145)	<0.001
aBMC	0.067 (–0.025, 0.158)	0.152	0.0004 (–0.091, .090)	0.993	0.051 (–0.046, 0.154)	0.293	0.021 (–0.077, 0.118)	0.677

CI = confidence interval; BMD = bone mineral density; CSMI = cross‐sectional moment of inertia (cm^4^); BA = bone area; BMC = bone mineral content; aBMC = area‐adjusted total body bone mineral content.

Table shows results of linear regression analysis between hip and total body DXA measurements and arterial distensibility in 3366 individuals. Results are standard deviation change in arterial distensibility per standard deviation increase in exposure (95% confidence intervals) and *p* value. Model 1 = unadjusted analysis; model 2 = adjustment for age; model 3 = model 2 plus additional adjustment for height, lean mass, fat mass, menopause, smoking, hormone replacement, calcium and/or vitamin D supplement use, and education.

Total body BMD, BMC, and BA were positively associated with arterial distensibility (model 1) (Table 3). These associations were partially attenuated after age adjustment (model 2) but strengthened after further adjustment for model 3. Further adjustment for cIMT made little impact on these results.

#### Sensitivity analyses

When the associations between hip BMD, TB BMD, BMC, BA, aBMC, and cIMT were further adjusted for alcohol consumption, PA levels, calcium intake, and SEP (*n* = 1936), associations were similar to those observed in the whole group analysis (model 3). Furthermore, we saw similar results for the associations between hip and TB DXA measurements and arterial distensibility (Supplemental Tables S2 and S3).

When analyses were restricted to premenopausal women (*n* = 2382), the associations between hip BMD, TB BMD, BMC, BA, aBMC, and cIMT were unchanged compared with the whole group analysis (*n* = 3366) (Supplemental Table S4). In terms of arterial distensibility, the association with total hip BMD (model 3; 0.069 [0.025, 0.113]; *p* = 0.002), TB BMD, BMC, and BA were all stronger when analyses were restricted to premenopausal women alone (Supplemental Table S5).

### Adolescents

Having identified positive relationships between bone parameters and cIMT in middle‐aged women, we wanted to determine whether similar patterns are also found at younger ages. Table 4 shows the characteristics in our adolescent study population. Their mean (SD) age was 17.8 (0.38) years. A total of 4368 (1944 males; 2424 females) had data available for bone measurements and cIMT. Height, lean mass, and cIMT were greater in males, whereas fat mass was greater in females. Total hip BMD and BMC, and TB BMD and BMC were greater in males compared with females. Compared with adolescents who did not participate and/or had missing data, those with data available were more likely to be white and female (Supplemental Table S6).

**Table 4:** Characteristics of Participating ALSPAC Study Adolescents

	Mean (SD) for continuously measured variables and prevalence (%) for categorical variables						
	*n*	All	*n*	Males	*n*	Females	*p* Value^a^
Exposure measurements							
Total hip BMD (g/cm^2^)	4368	1.11 (0.15)	1944	1.17 (0.15)	2424	1.05 (0.12)	<0.001
Total hip BMC (g)	4368	38.4 (10.2)	1944	46.4 (8.92)	2424	32.0 (5.44)	<0.001
Femoral neck BMD (g/cm^2^)	4368	1.09 (0.14)	1944	1.14 (0.15)	2424	1.05 (0.12)	<0.001
Trochanter BMD (g/cm^2^)	4368	0.88 (0.14)	1944	0.94 (0.14)	2424	0.83 (0.12)	<0.001
CSMI (cm^4^)	4368	2.07 (0.83)	1944	2.72 (0.75)	2424	1.56 (0.45)	<0.001
TB BMD minus head (g/cm^2^)	4368	1.09 (0.10)	1944	1.14 (0.10)	2424	1.04 (0.08)	<0.001
TB BMC minus head (g)	4368	2280.3 (489.8)	1944	2567.0 (463.5)	2424	2050.3 (375.7)	<0.001
TB area minus head (cm^2^)	4368	2078.7 (281.8)	1944	2228.2 (247.6)	2424	1958.8 (248.3)	<0.001
Outcome measurement							
cIMT (mm)	4368	0.48 (0.04)	1944	0.48 (0.05)	2424	0.47 (0.04)	<0.001
Confounder variables							
Age (years)	4368	17.8 (0.38)	1944	17.8 (0.37)	2424	17.8 (0.39)	0.273
Height (cm)	4368	171.3 (9.26)	1944	178.7 (6.61)	2424	165.3 (6.25)	<0.001
Log_e_ fat mass (kg)	4368	2.74 (0.60)	1944	2.42 (0.64)	2424	2.99 (0.41)	<0.001
Lean mass (kg)	4368	45.7 (9.95)	1944	55.1 (6.19)	2424	38.1 (4.28)	<0.001
Current smoker [*n* (%)]^b^	2836	476 (16.8)	1271	194 (15.3)	1565	282 (18.0)	0.051
Frequency of alcohol consumption [*n* (%)]^b^	2836						<0.001
Never or ≤monthly		787 (27.8)		299 (23.5)		488 (31.2)	
2–3 times/month		1335 (47.0)		595 (46.8)		740 (47.3)	
≥2/week		714 (25.2)		377 (29.7)		337 (21.5)	
Maternal social class [*n* (%)]^b^	2836						0.055
I		243 (8.6)		117 (9.2)		126 (8.1)	
II		1047 (36.9)		494 (38.9)		553 (35.3)	
III (non‐manual)		1114 (39.3)		495 (39.0)		619 (39.5)	
III (manual		176 (6.2)		73 (5.7)		103 (6.6)	
IV/V		256 (9.0)		92 (7.2)		164 (10.5)	
Other variables							
BMI	4362	22.8 (4.1)	1942	22.8 (4.1)	2420	23.0 (4.2)	0.001

SD = standard deviation; cIMT = common carotid intima‐media thickness; TB = total body; CSMI = cross‐sectional moment of inertia (cm^4^); BMD = bone mineral density; BMC = bone mineral content.

^a^ Unpaired *t* test for continuous variables and chi‐square test for categorical variables to assess the null hypothesis of no difference between males and females.

^b^ Valid data available only in a subgroup, *n* = 2836.

#### Association between hip and total body DXA measurements and cIMT (Table 5)

Hip DXA measurements, including total hip, femoral neck and trochanteric BMD, hip BMC, and TB DXA measurements, including TB BMD, BMC, and BA, were all positively associated with cIMT (model 1) (Table 5). These results were partially attenuated after age and sex adjustment (model 2). Additional adjustment for height and lean and fat mass led to further, but by no means complete, attenuation. A positive association was found between hip strength (CSMI) and cIMT (model 1; 0.164 [0.135, 0.193]; *p* < 0.001), which was fully attenuated after full confounder adjustment (model 3; –0.018 [–0.071, 0.036]; *p* = 0.517). Similarly, we saw no evidence of an association between aBMC and cIMT in our fully adjusted model (model 3; 1.02 [–0.010, 0.204]; *p* = 0.074).

**Table 5:** The Association Between Hip and Total Body DXA Measurements and cIMT Among ALSPAC Study Adolescents

	SD change in cIMT per 1 SD change in exposure (95% CI) in males and females combined (*n* = 4368)					
Exposure	Model 1	*p* Value	Model 2	*p* Value	Model 3	*p* Value
Total hip BMD	0.166 (0.137, 0.196)	<0.001	0.124 (0.092, 0.156)	<0.001	0.072 (0.035, 0.110)	<0.001
Total hip BMC	0.199 (0.170, 0.229)	<0.001	0.178 (0.137, 0.220)	<0.001	0.089 (0.031, 0.147)	0.003
Femoral neck BMD	0.152 (0.123, 0.182)	<0.001	0.116 (0.085, 0.147)	<0.001	0.067 (0.031, 0.102)	<0.001
Trochanter BMD	0.170 (0.140, 0.199)	<0.001	0.128 (0.096, 0.160)	<0.001	0.080 (0.043, 0.117)	<0.001
CSMI	0.164 (0.135, 0.193)	<0.001	0.108 (0.067, 0.148)	<0.001	–0.013 (–0.066,0.040)	0.622
Total body BMD	0.177 (0.147, 0.206)	<0.001	0.131 (0.097, 0.166)	<0.001	0.072 (0.025, 0.118)	0.002
Total body BA	0.180 (0.150, 0.209)	<0.001	0.112 (0.078, 0.145)	<0.001	0.104 (0.042, 0.165)	0.001
Total body BMC	0.161 (0.131, 0.190)	<0.001	0.135 (0.100, 0.169)	<0.001	0.115 (0.055, 0.175)	<0.001
aBMC	0.276 (0.181, 0.370)	<0.001	0.213 (0.115, 0.311)	<0.001	0.102 (–0.010, 0.214)	0.074

cIMT = common carotid intima‐media thickness; CI = confidence interval; BMD = bone mineral density; BMC = bone mineral content; CSMI = cross‐sectional moment of inertia (cm^4^).

Table shows results of linear regression analysis between hip DXA measurements and cIMT in 4368 individuals. Results are standard deviation change in cIMT per standard deviation increase in exposure (95% confidence intervals) and *p* value. Model 1 = unadjusted analysis; model 2 = adjustment for age and sex; model 3 = model 2 plus additional adjustment for height and lean and fat mass.

Results for males and females were similar (Supplemental Tables S7 and S8), and we detected no evidence to support a sex interaction with any exposure variable (*p* values for interaction >0.1).

#### Sensitivity analyses

When the associations between hip BMD and BMC, femoral neck and trochanteric BMD, CSMI, TB BMD, BA, and aBMC, and cIMT were further adjusted for alcohol consumption, smoking, and SEP (*n* = 2836), associations were similar to those observed in the whole group analysis (model 3) (Supplemental Table S9).

## Discussion

We examined the relationship between a range of bone parameters and preclinical atherosclerosis as measured by cIMT and arterial distensibility, in a population‐based study of 3366 women. In contrast to most previous studies, we examined these relationships in relatively young individuals, who were predominantly premenopausal women. We found that hip and total body DXA measurements were positively asociated with cIMT, though these associations were all relatively weak with adjusted correlation coefficients in the range of 0.047 to 0.071 and 0.061 to 0.118 for hip and total body DXA measurements, respectively.

Our findings are consistent with one previous cross‐sectional study of 535 women and 335 men (aged 18 to 97 years), which showed a weak positive association between hip BMD and cIMT, but only in women aged <60 years, and a clearer positive association between ultradistal radius BMD and cIMT in men, again aged <60 years.^5^ In contrast, other published cross‐sectional and prospective studies, albeit in older postmenopasual women, have reported greater cIMT in women with osteoporosis compared with those with normal bone mass,^21^ a negative correlation between cIMT and BMD,^22^ an increased prevalence of carotid atherosclerosis in women with low BMD and high osteocalcin levels,^23^ and inverse associations of CVD with BMD, osteoporosis, and fractures.^2^ Given our contrasting findings, we went on to examine associations between bone parameters and a functional marker of CVD risk, as measured by arterial distensibility. We found that greater femoral neck BMD, hip strength, and TB DXA measurements were all associated with reduced arterial stiffness, consistent with previous published findings in postmenopausal women associating low BMD with increased arterial stiffness.^24^

Whereas our observed associations between BMD and arterial distensibility are consistent with those previously reported for osteoporosis and cardiovascular disease, we hypothesize that the assocation we saw with cIMT may reflect a separate relationship between BMD and vascular size, which is evident in younger populations before the onset of significant atherosclerosis. To explore this possibility, we examined associations in a second population comprising 4368 adolescents (children of the women we analysed first). We observed a similar positive association between hip and total body DXA measurements and cIMT in these adolescents to those found in the mothers’ cohort.

The suggestion that cIMT measurements in younger individuals reflect vascular size independently of atherosclerosis, in contrast to arterial distensibility, is consistent with a previous study conducted in children and adolescents that found that cIMT increased with height, whereas carotid artery distensibility was largely independent.^25^ BMD reaches a peak in early adulthood and generally remains constant until the menopausal transition, after which it declines.^26^ In contrast, cIMT increases linearly with age.^27^, ^28^ Hence, the positive relationship between bone size and arterial thickness that we observed in premenopausal women and their adolescent offspring could theoretically reflect shared processes involved in growth and maturation. Consistent with this suggestion, bone and vascular development share several common processes. For example, similar collagenous fibers are present in both bone and arteries, hence genetic collagen disorders can manifest both skeletal and arterial pathologies (e.g., Ehlers‐Danlos syndrome). A series of factors have now been identified regulating connective tissue development in both the skeleton and vascular tissues.^29^ Cathepsin K, as well as being a lysosomal product of osteclasts with a key role in elastin and collagen degradation in bone,^30^ also plays a role in degrading vascular elastin, where overactivity leads to arterial aneurysm formation.^31^ In adult men and women, heritability studies have proposed shared genetic architecture underlying bone and vascular development.^32^ Hence, since bone and vascular development share certain biological mechanisms, the positive association between BMD and cIMT that we observed could reflect common constitutive influences on these traits.

Conceivably, the relationship between BMD and arterial distensibility that we observed in ALSPAC mothers, and was found to be independent of cIMT, could reflect a shared association between bone and other components of vascular development. That arterial distensibility also reflects a constitutive characteristic related to vascular development in this age group, rather than a marker of early atherosclerosis, is consistent with our finding that the relationship with BMD was stronger in younger, premenopausal women. According to this view, one would expect to find a similar relationship between BMD and arterial distensibility in ALSPAC adolescents; however, we were unable to assess this because arterial distensibility data were not collected among adolescents. Vasculature development is one of the first steps in embryonic development, resulting in a complex network of arteries, capillaries, and veins. New blood vessels are formed de novo from angioblastic stem cells (a process called vasculognesis) and via expansion of existing vessels through pruning and vessel enlargement (angiogenesis).^33^ Endothelial cell growth is regulated by vascular endothelial growth factor (VEGF), which also acts as an essential mediator during endochondrial ossification and intramembranous ossification. VEGF is involved in many processes, including osteoblast differentiation and recruitment of osteoclasts, and previous evidence suggests joint contribution of angiogenesis and bone formation during skeletal development.^34^, ^35^

We examined healthy populations of premenopausal women and adolescents with mean cIMT values of 0.56 mm (range 0.39 to 0.96) and 0.48 mm (range 0.34 to 0.69), respectively. Our measures are consistent with pubished normal mean (±SD) values for cIMT in adolescents (aged 17 to 20 years), range between 0.39 mm ± 0.03 and 0.40 mm ± 0.03 in males and females, respectively,^36^ and among adults overall 0.49 mm ± 0.06 (aged 35 to 39 years), 0.52 mm ± 0.06 (aged 40 to 49 years) and 0.56 ± 0.08 (aged 50 to 59 years).^37^ cIMT, aside from increasing with age (by 0.01 to 0.03 mm per year^38^), has been positively associated with systolic BP (0.0025‐mm increase in cIMT per 1‐mm Hg increase in systolic BP), smoking (0.0175‐mm increase in cIMT in smokers compared with non‐smokers), and diabetes, while being negatively associated with HDL cholesterol.^39^

### Limitations

Loss to follow‐up may have introduced selection bias if the associations are different in those who were not included and will have reduced statistical efficiency. However, in previous work, we have shown that loss to follow‐up is unlikely to result in major bias if observed variables that differ between those included and excluded are adjusted for as we have done here.^40^ Although we adjusted for a number of confounders, residual confounding could explain the persisting associations that we have found; in particular, our measurements of smoking and PA are relatively inprecise and prone to reporting error. This study is cross‐sectional, and although we have presented the associations with BMD as the exposure and cIMT as the outcome, some other studies conceptualize the relationship in the opposite direction. It is not possible to delineate the direction of causality using this design, nor in fact whether there is a true causal relationship in either direction, as correlations could exist because of residual confounding, including by shared biological pathways or genetic eitiology; e.g., osteoprotegerin gene variants have been found to be associated with both BMD^41^ and cIMT.^42^ Finally, cIMT, although a sensitive and reproducible technique for indentification of subclinical atherosclerosis (previously reported coefficients of variation vary between 5.4%^43^ and 3.1% and 7.8% in experienced and novice readers, respectively^44^), it remains a research tool and is unlikely to provide useful clinical information about BMD.

### Conclusion

We investigated associations between hip and whole body BMD and preclinical markers of atherosclerosis risk in ALSPAC mothers and adolescent offspring. We observed positive associations between BMD and cIMT, to a similar extent in mothers and adolescents, and a positive association between BMD and arterial distensibility, strongest in premenopausal women. These associations persisted after adjustment for a range of possible confounders. Rather than a relationship with preclinical atherosclerosis, in these relatively young populations, we speculate that observed associations between BMD, cIMT, and arterial distensibility may reflect a shared relationship between bone and vascular growth and development.

## Disclosures

All authors state that they have no conflicts of interest.

## Supporting information

Supporting Information.
