# Supplementary material for: Bone Mineral Density Is Positively Related to Carotid Intima‐Media Thickness: Findings From a Population‐Based Study in Adolescents and Premenopausal Women
Source: J Bone Miner Res. 2016 Sep 1;31(12):2139–48. doi: 10.1002/jbmr.2903 (PMC5244498; doi:10.1002/jbmr.2903)
Supplement: Supplementary file 1 — Supporting Information. [file JBMR-31-2139-s001.docx]

**Supplementary tables and figures for**

**Title:** Bone mineral density is positively related to carotid intima-media thickness: findings from a population based study in adolescents and pre-menopausal women

**Authors:** Monika Frysz, Kevin Deere, Debbie A. Lawlor, Li Benfield, Jon H. Tobias, Celia L. Gregson

**Table of Contents**

[**Supplementary Table 1** Baseline characteristics in women who attended the research clinic, and had data available for exposure, outcome and main confounder variables, and women initially recruited but did not attend the first follow up clinic assessment of mothers 2](#_Toc440543461)

[**Supplementary Table 2** Sensitivity analysis for the association between total hip BMD, total body BMD, total body BMC and total bone area and common carotid intima-media thickness (mm) in a subgroup of ALSPAC study women who had complete data for all potential confounders; (N= 1,936) 3](#_Toc440543462)

[**Supplementary Table 3** Sensitivity analysis for the association between total hip BMD, total body BMD, total body BMC and total bone area and mean arterial distensibility (mm) in a subgroup of ALSPAC study women who had complete data for all potential confounders; (N= 1,936) 4](#_Toc440543463)

[**Supplementary Table 4** Sensitivity analysis for the association between total hip BMD, total body BMD, total body BMC and total bone area and common carotid intima-media thickness (mm) restricted to premenopausal women (n= 2,382) 5](#_Toc440543464)

[**Supplementary Table 5** Sensitivity analysis for the association between total hip BMD, total body BMD, total body BMC and total bone area and mean arterial distensibility (mm) restricted to premenopausal women (n= 2,382) 6](#_Toc440543465)

[**Supplementary Table 6** A comparison of characteristics between adolescents who did and did not participate in the assessment clinic 7](#_Toc440543466)

[**Supplementary Table 7** The association between hip DXA measurements and common carotid intima-media thickness (mm) amongst ALSPAC study adolescents stratified by gender 8](#_Toc440543467)

[**Supplementary Table 8** The association between TB DXA measurements and common carotid intima-media thickness (mm) amongst ALSPAC study adolescents stratified by gender 9](#_Toc440543468)

[**Supplementary Table 9** Sensitivity analysis for the association between total hip and TB DXA measurements and common carotid intima-media thickness (mm) in a subgroup of ALSPAC study adolescents who had complete data for all potential confounders; (N= 2,836) 10](#_Toc440543469)

[**Supplementary figure 1** Flow diagram showing the distribution of participants from the recruitment to the present study population consisting of 3,366 mothers and 4,368 adolescents with complete data used for main analyses. 11](#_Toc440543470)

**Supplementary Table 1** Baseline characteristics in women who attended the research clinic, and had data available for exposure, outcome and main confounder variables, and women initially recruited but did not attend the first follow up clinic assessment of mothers

|  | Mean (SD) for continuous variables and prevalence n(%) for categorical variables | | | | |
| --- | --- | --- | --- | --- | --- |
|  | Subjects with no data available N = 10,395 | | Current study population N = 3,366 | |  |
|  | N |  | N |  | P-value^1^ |
| Age at birth of child (years) | 10,381 | 27.3 (5.0) | 3,366 | 30.0 (4.3) | <0.001 |
| Pre-pregnancy BMI (kg/m^2^) | 8,206 | 23.1 (4.0) | 3,154 | 22.5 (3.2) | <0.001 |
| Had a university degree at time of index pregnancy [n (%)] | 8,923 | 904 (10.1) | 3,366 | 675 (20.1) | <0.001 |
| Ever smoked [n (%)] | 9,478 | 5,223 (55.1) | 3,363 | 1,291 (38.4) | <0.001 |
| Owned property at time of index pregnancy [n (%)] | 9,505 | 6,466 (68.0) | 3,311 | 2,927 (88.4) | <0.001 |

^1^Unpaired t-test for continuous variables and chi-square test for categorical variables to assess the null hypothesis of no difference in those who did ad those who did not attend the follow-up clinic.

# **Supplementary Table 2** Sensitivity analysis for the association between total hip BMD, total body BMD, total body BMC and total bone area and common carotid intima-media thickness (mm) in a subgroup of ALSPAC study women who had complete data for all potential confounders; (N= 1,936)

|  | SD change in cIMT per 1 SD change in exposure (95% CI) (n= 1,936) | | | | | |
| --- | --- | --- | --- | --- | --- | --- |
| **Exposure** | **Crude** | **p value** | **Adjusted^a^** | **p value** | **Adjusted^b^** | **p value** |
| Total hip BMD | 0.054 (0.007, 0.102) | 0.024 | 0.052 (0.0004, 0.104) | 0.048 | 0.057 (0.004, 0.109) | 0.034 |
| Total body BMD | 0.073 (0.027, 0.120) | 0.002 | 0.072 (0.017, 0.127) | 0.011 | 0.076 (0.020, 0.132) | 0.007 |
| Total body BMC | 0.080 (0.034, 0.127) | 0.001 | 0.078 (0.011, 0.145) | 0.023 | 0.082 (0.014, 0.150) | 0.018 |
| Total body BA | 0.070 (0.023, 0.117) | 0.004 | 0.049 (-0.022, 0.121) | 0.175 | 0.051 (-0.021, 0.123) | 0.169 |
| aBMC | 0.120 (-0.007, 0.246) | 0.064 | 0.155 (0.019, 0.291) | 0.025 | 0.166 (0.029, 0.304) | 0.018 |

Abbreviations: cIMT, common carotid intima-media thickness; BMD, bone mineral density; BA, bone area; BMC, bone mineral content; aBMC, area-adjusted bone mineral content. ^a^ Adjusted for age, height, lean mass, fat mass, menopause, smoking, hormone replacement, calcium and/or vitamin D supplement use and education (model 3 in main analyses); ^b^ the same as ^a^ plus additional adjustment for alcohol consumption, physical activity level, calcium intake and socio-economic position

**Supplementary Table 3** Sensitivity analysis for the association between total hip BMD, total body BMD, total body BMC and total bone area and mean arterial distensibility (mm) in a subgroup of ALSPAC study women who had complete data for all potential confounders; (N= 1,936)

|  | SD change in average arterial distensibility per 1 SD change in exposure (95% CI) (n= 1,936) | | | | | |
| --- | --- | --- | --- | --- | --- | --- |
| **Exposure** | **Crude** | **p value** | **Adjusted^a^** | **p value** | **Adjusted^b^** | **p value** |
| Total hip BMD | 0.052 (0.007, 0.097) | 0.023 | 0.053 (0.004, 0.103) | 0.034 | 0.053 (0.003, 0.102) | 0.037 |
| Total body BMD | 0.096 (0.053, 0.140) | <0.001 | 0.097 (0.045, 0.149) | <0.001 | 0.095 (0.042, 0.148) | <0.001 |
| Total body BMC | 0.098 (0.054, 0.141) | <0.001 | 0.125 (0.061, 0.189) | <0.001 | 0.120 (0.056, 0.185) | <0.001 |
| Total body BA | 0.082 (0.038, 0.127) | <0.001 | 0.105 (0.037, 0.173) | 0.002 | 0.099 (0.030, 0.167) | 0.005 |
| aBMC | 0.158 (0.038, 0.278) | 0.010 | 0.162 (0.033, 0.292) | 0.014 | 0.160 (0.030, 0.290) | 0.016 |

Abbreviations: BMD, bone mineral density; BA, bone area; BMC, bone mineral content; aBMC, area-adjusted bone mineral content.
 ^a^ Adjusted for age, height, lean mass, fat mass, menopause, smoking, hormone replacement, calcium and/or vitamin D supplement use and education (model 3 in main analyses); ^b^ the same as ^a^ plus additional adjustment for alcohol consumption, physical activity level, calcium intake and socio-economic position

# **Supplementary Table 4** Sensitivity analysis for the association between total hip BMD, total body BMD, total body BMC and total bone area and common carotid intima-media thickness (mm) restricted to premenopausal women (n= 2,382)

|  | SD change in cIMT per 1 SD change in exposure (95% CI) (n= 2,382) | | | | | |
| --- | --- | --- | --- | --- | --- | --- |
| **Exposure** | **Model 1** | **p value** | **Model 2** | **p value** | **Model 3** | **p value** |
| Total hip BMD | 0.098 (0.058, 0.139) | <0.001 | 0.110 (0.071, 0.150) | <0.001 | 0.068 (0.024, 0.112) | 0.002 |
| Total body BMD | 0.115 (0.074, 0.156) | <0.001 | 0.123 (0.083, 0.163) | <0.001 | 0.066 (0.017, 0.114) | 0.008 |
| Total body BA | 0.102 (0.062, 0.143) | <0.001 | 0.122 (0.082, 0.161) | <0.001 | 0.078 (0.016, 0.140) | 0.013 |
| Total body BMC | 0.119 (0.079, 0.160) | <0.001 | 0.136 (0.097, 0.176) | <0.001 | 0.091 (0.033, 0.150) | 0.002 |
| aBMC | 0.189 (0.077, 0.300) | 0.001 | 0.178 (0.069, 0.287) | 0.001 | 0.111 (-0.008, 0.230) | 0.068 |

Abbreviations: cIMT, common carotid intima-media thickness; BMD, bone mineral density; BA, bone area; BMC, bone mineral content; aBMC, area-adjusted bone mineral content. Table shows results of linear regression analysis between bone measurements and cIMT in 2933 individuals. Results are standard deviation change in cIMT per standard deviation increase in exposure (95% confidence intervals) and P value. Model 1=unadjusted analysis, model 2 = adjustment for age; model 3 = model 2 plus additional adjustment for height, lean mass, fat mass, menopause, smoking, hormone replacement, calcium and/or vitamin D supplement use and education

# **Supplementary Table 5** Sensitivity analysis for the association between total hip BMD, total body BMD, total body BMC and total bone area and mean arterial distensibility (mm) restricted to premenopausal women (n= 2,382)

|  | SD change in average arterial distensibility per 1 SD change in exposure (95% CI) (n= 2,382) | | | | | |
| --- | --- | --- | --- | --- | --- | --- |
| **Exposure** | **Model 1** | **p value** | **Model 2** | **p value** | **Model 3** | **p value** |
| Total hip BMD | 0.041 (0.001, 0.082) | 0.047 | 0.031 (-0.009, 0.071) | 0.126 | 0.069 (0.025, 0.113) | 0.002 |
| Total body BMD | 0.068 (0.027, 0.108) | 0.001 | 0.061 (0.021, 0.101) | 0.003 | 0.099 (0.050, 0.148) | <0.001 |
| Total body BA | 0.077 (0.036, 0.118) | <0.001 | 0.062 (0.021, 0.102) | 0.003 | 0.137 (0.075, 0.199) | <0.001 |
| Total body BMC | 0.082 (0.042, 0.123) | <0.001 | 0.069 (0.029, 0.109) | <0.001 | 0.149 (0.090, 0.208) | <0.001 |
| aBMC | 0.080 (-0.032, 0.193) | 0.161 | 0.89 (-0.021, 0.200) | 0.114 | 0.151 (0.031, 0.270) | 0.014 |

Abbreviations: BMD, bone mineral density; BA, bone area; BMC, bone mineral content; aBMC, area-adjusted bone mineral content. Table shows results of linear regression analysis between bone measurements and cIMT in 2933 individuals. Results are standard deviation change in cIMT per standard deviation increase in exposure (95% confidence intervals) and P value. Model 1=unadjusted analysis, model 2 = adjustment for age; model 3 = model 2 plus additional adjustment for height, lean mass, fat mass, menopause, smoking, hormone replacement, calcium and/or vitamin D supplement use and education

# **Supplementary Table 6** A comparison of characteristics between adolescents who did and did not participate in the assessment clinic

| Characteristic | Category | Mean (SD) for continuous variables and prevalence n(%) for categorical variables | | | | |
| --- | --- | --- | --- | --- | --- | --- |
|  |  | Subjects with no data available N = 10,333 | | Current study population N = 4,368 | |  |
|  |  | N |  | N |  | P-value^1^ |
| Child sex | Male | 10,316 | 5,592 (54.2) | 4,368 | 1,944 (44.5) | <0.001 |
| Child ethnicity | White | 8,170 | 7,735 (94.7) | 3,907 | 3,733 (95.6) | 0.041 |

# **Supplementary Table 7** The association between hip DXA measurements and common carotid intima-media thickness (mm) amongst ALSPAC study adolescents stratified by gender

|  | SD change in cIMT per 1 SD change in exposure (95% CI) | | | | | |
| --- | --- | --- | --- | --- | --- | --- |
|  | **Males N=1,944** |  |  |  |  |  |
| Exposure | **Model 1** | **p value** | **Model 2** | **p value** | **Model 3** | **p value** |
| Total hip BMD | 0.140 (0.096, 0.184) | <0.001 | 0.140 (0.096, 0.184) | <0.001 | 0.087 (0.033, 0.141) | 0.002 |
| Total hip BMC | 0.169 (0.118, 0.221) | <0.001 | 0.169 (0.118, 0.221) | <0.001 | 0.091 (0.016, 0.166) | 0.018 |
| Femoral neck BMD | 0.127 (0.083, 0.170) | <0.001 | 0.127 (0.083, 0.170) | <0.001 | 0.073 (0.021, 0.125) | 0.006 |
| Trochanter BMD | 0.143 (0.099, 0.187) | <0.001 | 0.143 (0.099, 0.187) | <0.001 | 0.095 (0.041, 0.148) | <0.001 |
| CSMI | 0.112 (0.061, 0.162) | <0.001 | 0.112 (0.061, 0.162) | <0.001 | -0.002(-0.072, 0.067) | 0.945 |
|  | **Females 2,424** |  |  |  |  |  |
|  | **Model 1** | **p value** | **Model 2** | **p value** | **Model 3** | **p value** |
| Total hip BMD | 0.104 (0.058, 0.151) | <0.001 | 0.104 (0.058, 0.151) | <0.001 | 0.058 (0.006, 0.110) | 0.029 |
| Total hip BMC | 0.198 (0.127, 0.269) | <0.001 | 0.198 (0.127, 0.269) | <0.001 | 0.102 (0.005, 0.198) | 0.039 |
| Femoral neck BMD | 0.104 (0.060, 0.148) | <0.001 | 0.104 (0.060, 0.148) | <0.001 | 0.061 (0.012, 0.110) | 0.015 |
| Trochanter BMD | 0.109 (0.063, 0.156) | <0.001 | 0.109 (0.063, 0.156) | <0.001 | 0.065 (0.013, 0.117) | 0.014 |
| CSMI | 0.099 (0.028, 0.170) | 0.007 | 0.099 (0.028, 0.170) | 0.007 | -0.018 (-0.107,0.071) | 0.693 |
|  | **Males + Females N= 4,368** |  |  |  |  |  |
|  | **Model 1** | **p value** | **Model 2** | **p value** | **Model 3** | **p value** |
| Total hip BMD | 0.166 (0.137, 0.196) | <0.001 | 0.166 (0.137, 0.196) | <0.001 | 0.080 (0.044, 0.117) | <0.001 |
| Total hip BMC | 0.199 (0.170, 0.229) | <0.001 | 0.199 (0.170, 0.229) | <0.001 | 0.093 (0.035, 0.151) | 0.002 |
| Femoral neck BMD | 0.152 (0.123, 0.182) | <0.001 | 0.152 (0.123, 0.182) | <0.001 | 0.075 (0.041, 0.110) | <0.001 |
| Trochanter BMD | 0.170 (0.140, 0.199) | <0.001 | 0.170 (0.140, 0.199) | <0.001 | 0.087 (0.051, 0.124) | <0.001 |
| CSMI | 0.164 (0.135, 0.193) | <0.001 | 0.164 (0.135, 0.193) | <0.001 | -0.018 (-0.071,0.036) | 0.517 |

Abbreviations: cIMT, common carotid intima-media thickness; CI, confidence interval; BMD, bone mineral density; BMC, bone mineral content; CSMI, cross-sectional moment of inertia (cm^4^). Table shows results of linear regression analysis between hip DXA measurements and cIMT in 4,368 individuals (1,944 males and 2,424 females). Results are standard deviation change in cIMT per standard deviation increase in exposure (95% confidence intervals) and p value. Model 1=unadjusted analysis, model 2 = adjustment for age and gender; model 3 = model 2 plus additional adjustment for height, lean and fat mass.

# **Supplementary Table 8** The association between TB DXA measurements and common carotid intima-media thickness (mm) amongst ALSPAC study adolescents stratified by gender

|  | SD change in cIMT per 1 SD change in exposure (95% CI) | | | | | |
| --- | --- | --- | --- | --- | --- | --- |
|  | **Males N=1,944** |  |  |  |  |  |
| Exposure | **Model 1** | **p value** | **Model 2** | **p value** | **Model 3** | **p value** |
| Total body BMD | 0.135 (0.088, 0.181) | <0.001 | 0.135 (0.088, 0.181) | <0.001 | 0.068 (0.003, 0.133) | 0.042 |
| Total body BA | 0.146 (0.098, 0.194) | <0.001 | 0.146 (0.098, 0.195) | <0.001 | 0.118 (0.028, 0.209) | 0.010 |
| Total body BMC | 0.135 (0.083, 0.187) | <0.001 | 0.135 (0.083, 0.187) | <0.001 | 0.111 (0.002, 0.221) | 0.047 |
| aBMC | 0.222 (0.091, 0.354) | 0.001 | 0.222 (0.091, 0.354) | 0.001 | 0.126 (-0.027, 0.278) | 0.105 |
|  | **Females 2,424** |  |  |  |  |  |
|  | **Model 1** | **p value** | **Model 2** | **p value** | **Model 3** | **p value** |
| Total body BMD | 0.127 (0.076, 0.178) | <0.001 | 0.127 (0.076, 0.178) | <0.001 | 0.082 (0.015, 0.148) | 0.017 |
| Total body BA | 0.120 (0.071, 0.170) | <0.001 | 0.121 (0.071, 0.170) | <0.001 | 0.148 (0.061, 0.234) | 0.001 |
| Total body BMC | 0.093 (0.050, 0.137) | <0.001 | 0.093 (0.050, 0.137) | <0.001 | 0.145 (0.063, 0.227) | 0.001 |
| aBMC | 0.193 (0.031, 0.355) | 0.020 | 0.193 (0.031, 0.355) | 0.020 | 0.059 (-0.119, 0.236) | 0.519 |
|  | **Males + Females N= 4,368** |  |  |  |  |  |
|  | **Model 1** | **p value** | **Model 2** | **p value** | **Model 3** | **p value** |
| Total body BMD | 0.177 (0.147, 0.206) | <0.001 | 0.177 (0.147, 0.206) | <0.001 | 0.081 (0.035, 0.126) | 0.001 |
| Total body BA | 0.180 (0.150, 0.209) | <0.001 | 0.180 (0.150, 0.209) | <0.001 | 0.123 (0.063, 0.183) | <0.001 |
| Total body BMC | 0.161 (0.131, 0.190) | <0.001 | 0.161 (0.131, 0.190) | <0.001 | 0.105 (0.043, 0.167) | 0.001 |
| aBMC | 0.276 (0.181, 0.370) | <0.001 | 0.276 (0.181, 0.370) | <0.001 | 0.127 (0.016, 0.238) | 0.025 |

Abbreviations: cIMT, common carotid intima-media thickness; CI, confidence interval; BMD, bone mineral density; BMC, bone mineral content; CSMI, cross-sectional moment of inertia (cm^4^). Table shows results of linear regression analysis between hip DXA measurements and cIMT in 4,368 individuals (1,944 males and 2,424 females). Results are standard deviation change in cIMT per standard deviation increase in exposure (95% confidence intervals) and p value. Model 1=unadjusted analysis, model 2 = adjustment for age and gender; model 3 = model 2 plus additional adjustment for height, lean and fat mass.

# **Supplementary Table 9** Sensitivity analysis for the association between total hip and TB DXA measurements and common carotid intima-media thickness (mm) in a subgroup of ALSPAC study adolescents who had complete data for all potential confounders; (N= 2,836)

|  | SD change in cIMT per 1 SD change in exposure (95% CI) in males and females combined (n=2,836) | | | | | |
| --- | --- | --- | --- | --- | --- | --- |
| **Exposure** | **Crude** | **p value** | **Adjusted^a^** | **p value** | **Adjusted^b^** | **p value** |
| Total hip BMD | 0.166 (0.129, 0.202) | <0.001 | 0.084 (0.038, 0.130) | <0.001 | 0.078 (0.032, 0.124) | 0.001 |
| Total hip BMC | 0.191 (0.155, 0.227) | <0.001 | 0.093 (0.022, 0.165) | 0.010 | 0.088 (0.017, 0.159) | 0.016 |
| Femoral neck BMD | 0.156 (0.119, 0.193) | <0.001 | 0.084 (0.040, 0.127) | <0.001 | 0.081 (0.037, 0.124) | <0.001 |
| Trochanter BMD | 0.171 (0.134, 0.207) | <0.001 | 0.093 (0.047, 0.138) | <0.001 | 0.087 (0.041, 0.132) | <0.001 |
| CSMI | 0.158 (0.122, 0.194) | <0.001 | -0.008 (-0.074, 0.057) | 0.804 | -0.011 (-0.077, 0.054) | 0.736 |
|  |  |  |  |  |  |  |
| Total body BMD | 0.168 (0.131, 0.204) | <0.001 | 0.070 (0.013, 0.126) | 0.016 | 0.066 (0.009, 0.122) | 0.023 |
| Total body BA | 0.148 (0.111, 0.185) | <0.001 | 0.093 (0.019, 0.168) | 0.014 | 0.091 (0.016, 0.166) | 0.017 |
| Total body BMC | 0.166 (0.130, 0.203) | <0.001 | 0.069 (-0.009, 0.147) | 0.082 | 0.071 (-0.007, 0.149) | 0.076 |
| aBMC | 0.279 (0.160, 0.398) | <0.001 | 0.127 (-0.016, 0.238) | 0.099 | 0.115 (-0.022, 0.252) | 0.099 |

Abbreviations: cIMT, common carotid intima-media thickness; BMD, bone mineral density; BMC, bone mineral content; CSMI, cross-sectional moment of inertia (cm^4^). Table shows results of linear regression analysis between hip DXA measurements and cIMT in 2,836 individuals (1,271 males and 1,565 females). Results are standard deviation change in cIMT per standard deviation increase in exposure (95% confidence intervals) and p value. **^a^** = adjusted for age, gender, height, lean and fat mass, ^b^ the same as ^a^ plus additional adjustment for alcohol consumption, smoking and socio-economic position.

Recruitment Phase II at age 7 years
Enrolled N = 452

Recruitment Phase III from age 8 years
Enrolled N = 254

Excluded 674 pregnancies:
69 unknown outcomes
604 no live-birth
1 live-birth from a twin pregnancy

Recruited pregnancies
N= 14, 541

Eligible pregnancies enrolled in ALSPAC
N = 15,247

Included pregnancy cohort
N= 13,867

Live- born children N = 14,775

Individual women recruited
N= 13,761

Alive at one year of age N = 14,701

Adolescents invited to follow-up research clinic

N= 10,101

Women invited to follow-up research clinic
N= 11,264

Adolescents attended follow-up research clinic

N= 5,217

Women attended research clinic
N= 4,834

2,836 adolescents with complete data on outcome, exposures and all potential confounders (including alcohol consumption, smoking and SEP)

4,368 adolescents with complete data on outcome, exposures and main confounders (height, lean and fat mass)

1,936 women with complete data on outcomes, exposures and all potential confounders (including alcohol consumption, physical activity, daily calcium intake and SEP)

3,366 women with complete data on outcomes, exposures and most confounders (except alcohol consumption, physical activity, daily calcium intake and SEP)

# **Supplementary figure 1** Flow diagram showing the distribution of participants from the recruitment to the present study population consisting of 3,366 mothers and 4,368 adolescents with complete data used for main analyses. cIMT, Carotid intima-media thickness; SEP, socio-economic position
